# Comparing GWAS Results of Complex Traits Using Full Genetic Model and Additive Models for Revealing Genetic Architecture

**DOI:** 10.1038/srep38600

**Published:** 2017-01-12

**Authors:** Md. Mamun Monir, Jun Zhu

**Affiliations:** 1 Institute of Bioinformatics, Zhejiang University, Hangzhou 310058, China

## Abstract

Most of the genome-wide association studies (GWASs) for human complex diseases have ignored dominance, epistasis and ethnic interactions. We conducted comparative GWASs for total cholesterol using full model and additive models, which illustrate the impacts of the ignoring genetic variants on analysis results and demonstrate how genetic effects of multiple loci could differ across different ethnic groups. There were 15 quantitative trait loci with 13 individual loci and 3 pairs of epistasis loci identified by full model, whereas only 14 loci (9 common loci and 5 different loci) identified by multi-loci additive model. Again, 4 full model detected loci were not detected using multi-loci additive model. PLINK-analysis identified two loci and GCTA-analysis detected only one locus with genome-wide significance. Full model identified three previously reported genes as well as several new genes. Bioinformatics analysis showed some new genes are related with cholesterol related chemicals and/or diseases. Analyses of cholesterol data and simulation studies revealed that the full model performs were better than the additive-model performs in terms of detecting power and unbiased estimations of genetic variants of complex traits.

Cholesterol is an important complex lipid contain sterol nucleus. Elevated level of cholesterol increased risk of cardiovascular disease including coronary heart disease, stroke and peripheral vascular disease. Moreover, it has also been linked to diabetes and high blood pressure. Several association studies with different approaches were previously performed to analyze cholesterol trait for a number of different populations^1^,^2^,^3^. For example, single-locus model to detect main effects, and two-locus model for epistasis were used to analyze cholesterol trait of Framingham Heart Study data^4^. Teslovich *et al*. reported 39 genome-wide significant loci related with total cholesterol (TC) through meta-analysis of several GWASs^5^. Most of the previous GWASs of cholesterol were individually tested for additive genetic effect of each locus and Bonferroni correction threshold of multiple tests had been used to determine genome-wide significance.

Usefulness of multi-loci approaches compared to single locus approaches were largely described in QTL mapping era^6^,^7^,^8^,^9^,^10^,^11^,^12^,^13^,^14^,^15^,^16^. In GWASs, multi-loci association studies also have better performance as compared to the single-locus association studies^16^,^17^,^18^. Multi-loci association studies may identify some genetic variants that jointly have significant effects but individually make only a small contribution^19^. Single locus mapping approaches might fail to detect loci due to lack of controlling genetic background variants. Popular single locus mixed model approaches use additive genetic relationship matrix to control genetic background and genetic relatedness. Yang *et al*. showed constructing genetic relationship matrix by excluding the chromosome that being tested, might improve analysis results^20^. However, genetic background control by using genetic relationship matrix is insufficient for large effects of several causal loci^17^. Again, single locus additive model approaches rarely explain more than a small proportion of the heritable variation^21^. Instead of individually testing the additive effects of loci, a full genetic model with genetic effects of multiple loci, epistasis and environment interaction can be analyze by linear mixed model approaches implemented in software *QTXNetwork*^22^.

In this study, we analyzed total cholesterol of multi-ethnic populations that including European-Americans (*E-A*), Chinese-Americans (*C-A*), African-Americans (*A-A*), and Hispanic-Americans (*H-A*). Four different association approaches were used: full model and multi-loci additive model through *QTXNetwork*, single locus linear mixed model approach through *GCTA*^23^, and single locus regression based approach through *PLINK*^24^. Repeated measures from two examinations (Exam-1: July 2000–July 2002; and Exam-3: January 2004–July 2005) were used as replications for full model and multi-loci additive model approaches to analyze total cholesterol of Multi-Ethnic Study of Atherosclerosis (MESA) population. Finally, we conducted simulations under two scenarios and by using perceived genetic architecture of cholesterol trait from full model and multi-loci additive model approaches to get possible explanations of differences in analyses results. This study presents usefulness of full model approach over additive model for complex trait analyses.

## Results

### Estimated heritability from full and additive model approaches

The estimated total heritability was 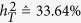 (Table 1) for detected quantitative trait SNPs (QTSs) through full model with their significant genetic and ethnic interaction effects, mostly due to epistasis effects (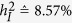), additive effects (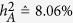), and dominance by ethnic interactions (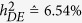). The estimated heritability due to non-additive effects was larger than estimated heritability due to additive effects only. We observed missing heritability for multi-loci additive model including only additive and additive by ethnic interaction effects (Table 1). Total estimated heritability for cholesterol using the multi-loci additive model was only 13.91%, which is less than half of the total heritability of full model.

### Genetic and ethnic specific effects of QTSs

**Figure 1:**
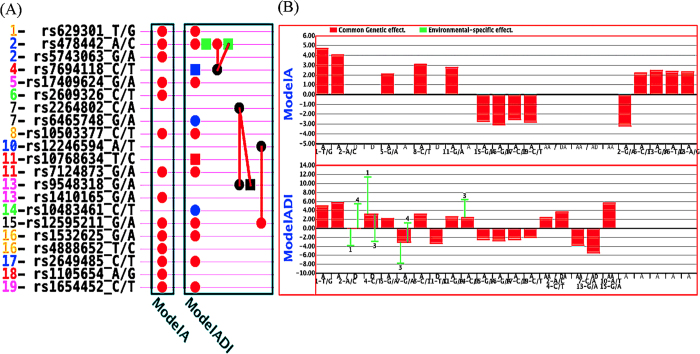
G × G and G × E plots of highly significant effects of QTSs detected from full and additive models. (**A**) G × G plot. Red circle dot: loci with additive effects; blue circle dot: loci with both additive and ethnic-specific effects; red square dot: loci with dominance effects; green square dot: loci with dominance × ethnic interaction effects; blue square dot: loci with both dominance and ethnic specific dominance effects; black dot with a line: loci with epistasis but no individual effect; Red line between two dot: *aa* or *ad* or *da* epistasis. (**B**) G × E plot. Loci were plotted by chromosome number and major/minor alleles; green line showing the ethnic specific effects of loci.

Two distinct steps were used to complete cholesterol data analyses. At first, we extracted a set of SNPs that were significantly identified through 1D, 2D and 3D scan by GMDR-GPU module and then used a linear mixed model approach to explore and build up an optimum model of genetic architecture of trait. Actually, the first step is a filtering step that assists to reduce computational time for further analysis. In the second step, we tested for experimental-wise statistical significance (α_*EW*_ = 0.05) of extracted set of SNPs using full model approach to reveal complex genetic architecture of trait. We plotted results of cholesterol data analyses by using full model and multi-loci additive model approaches in Fig. 1.

There were 15 QTSs (13 individual loci and 3 pairs of epistasis loci) identified for cholesterol with experimental-wise *P*-value (*P*_*EW*_) less than 1 × 10^−5^ using full model approach (Table 2, Fig. 1). Experimental-wise highly significant (−log_10_*P*_*EW*_ > 5) additive effects were detected for 11 QTSs. C/C of rs478442 near gene *APOB* (Apo lipoprotein B) had the most significant additive effect (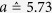, −log_10_*P*_*EW*_ > 47), while T/T of rs629301 in gene *CELSR2* had another most significant additive effect (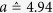, −log_10_*P*_*EW*_ > 34). In addition to additive effect, QTS rs629301 also had significant ethnic specific dominance effects negative for *E-A* population (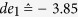) but positive for *H-A* population (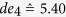). Gene CEL*SR2* located on chromosome 1p13.3 and its protein encodes a member of flamingo subfamily, part of cadherin superfamily; and *APOB* is a protein-coding gene, located at chromosome 2p24.1. Several variants near gene regions of *CELSR2-PSRC1-SORT1* and *APOB-TRDR15* have been reported to be associate with cholesterol in a number of previous GWASs^4^,^5^,^25^. *CELSR2* in the cholesterol gene cluster shows a significant association with coronary artery disease and its single nucleotide polymorphism regulates plasma cholesterol levels^25^. The QTS rs6465748 at 9.6 kb 5′ of *MYH16* had highly significant ethnic specific additive effects for African American (*A-A*) and Hispanic American (*H-A*) cohort in addition to its additive main effect. That implies the additive effect of QTS rs6465748 was significantly different in these two ethnic groups as compared to other two groups, whereas additive effect was the smallest for *A-A* population (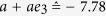) but the largest for *H-A* population (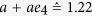). Heterozygote C/T of rs7694118 had significant dominance and dominance by ethnic interaction effects. Another locus T/C of rs10768634 had highly significant dominance effect only.

There were three pairs of QTSs identified with epistasis effects of additive × additive (*aa*), additive × dominance (*ad*), and dominance × additive (*da*). The significant additive × additive epistasis interaction (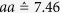, −log_10_*P*_*EW*_ > 13) was from A/A of rs12246594 in gene *SORCS1* × G/G of rs12595211 in gene *ETFA. SORCS1* encodes one family member of vacuolar protein sorting 10 (VPS10) domain-containing receptor proteins, located at chromosome 10q25.1. Variants of *SORCS1* gene have been reported associate with type-2 diabetes^26^,^27^, and Alzheimer’s diseases^28^,^29^, while these diseases were reported to associate with cholesterol^30^,^31^,^32^. *ETFA* is located on chromosome 15q24.3 that participates in catalyzing the initial step of the mitochondrial fatty acid beta-oxidation. Negative epistasis interactions between rs2264802 in gene *SKAP2* and rs9548318 in gene *UFM1* had highly significant additive × additive (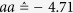, −log_10_*P*_*EW*_ > 26) and additive × dominance (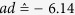, −log_10_*P*_*EW*_ > 12). *SKAP2* encodes protein contains an amino terminal coiled-coil domain for self-dimerization, a plecskstrin homology (PH) domain required for interactions with lipids at the membrane. Positive epistasis effects were detected between rs478442 near *APOB* and rs7694118 near *PCD*H10 (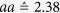 of A/A × C/C, 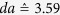 of A/C × C/C). The *APOB* gene is interact with the LDL receptor, and is fundamental for the regulation of plasma cholesterol in humans^33^. Genetic analysis of NMR-lipoprotein fractions in humans had shown the gene *PCDH10* related with LDL cholesterol^34^.

### Bioinformatics analysis of candidate genes corresponding to QTSs

**Figure 2:**
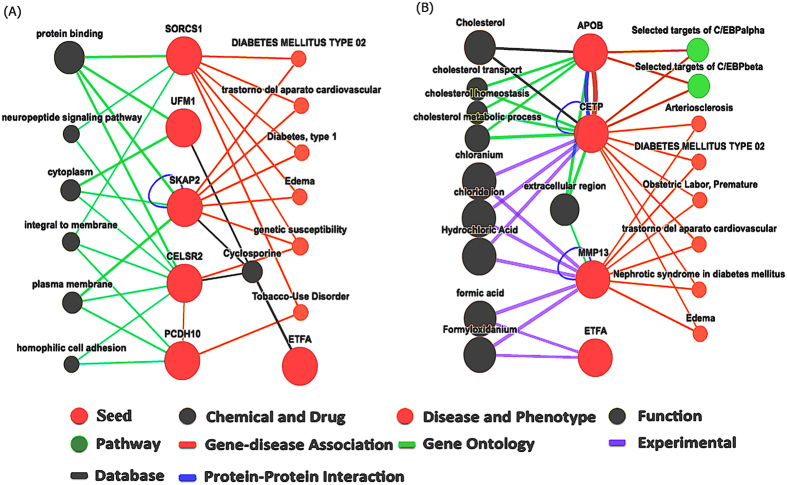
Bioinformatics analysis using *Biopubinfo* for set of detected genes. The size of balls and thickness of lines stand for number of related publications. Red balls represent seed genes; Orange balls represent association diseases; Olive balls represent associate functions; Red orange lines represent gene-disease association; Paris green lines represent gene ontology; Dark blue lines represent protein-protein interaction; Bronze lines represent pathway interaction; Dark gray lines represent database.

Bioinformatics analyses were applied by using *Biopubinfo* (http://ibi.zju.edu.cn/biopubinfo/) for candidate genes detected using full model approach. *Biopubinfo* is a search engine for tracing public biological information mainly covers data from life sciences and medical sciences, and includes a concept ontology database derived from several sources (UMLS, OLS, BioOntology.org). The candidate genes corresponding to detected QTSs were used as seeds for searching related pathway, functions, genes, chemical and drug information, protein-protein interactions, gene-disease association *etc*. Figure 2A shows relationship of four candidate genes (*CELSR2, SKAP2, ETFA* and *UFM1*) with chemical cyclosporine, whereas cyclosporine has significant relationship with blood cholesterol levels and cause of increasing blood cholesterol^35^.

There were relationships between genes *APOB* and *CETP* with cholesterol; genes *CETP* and *MMP13* with chemical chloranium, chloride ion and hydrochloric acid; genes *MMP13* and *ETFA* with formic acid and formyloxidanium (Fig. 2B). Gene-disease association reports the relationships of candidate genes with several diseases (Fig. 2), and the diseases might have relationship with cholesterol. Genes *SORCS1* and *SKAP2* have relationship with diabetes type 1, diabetes mellitus type 2, cardiovascular diseases, and Edema (Fig. 2A). Absorption and low synthesis of cholesterol might observes in patients with type-1 diabetic compared with non-diabetic control subjects^36^. The high-normal range with moderately elevated levels of total cholesterol and hemoglobin A1c defines a high-risk group for the progression to diabetic nephropathy and for clinical events related to arteriosclerotic cardiovascular disease^37^. Several experimental studies discussed about potential relationship of cholesterol with diabetes mellitus type-2^38^,^39^,^40^,^41^,^42^. Total cholesterol is the most important risk factor associated with clinically significant macular edema (CSME)^43^. Gene-disease association (Fig. 2A) shows *SORCS1, SKAP2*, and *CELSR2* genes are related with genetic susceptibility, and genes *SORCS1* and *PCDH10* are related with tobacco-use disorder. Protein–protein interaction was observed between *CELSR2* and *PCDH10*. Gene ontology search shows complex network among genes in terms of function, *e.g. SORCS1, UFM1*, and *CELSR2* are related through protein binding; *SORCS1, CELSR2, PCDH10* are related through integral to membrane etc. (Fig. 2).

### Association study with additive genetic models

Association analysis with full model identified several significant dominance and epistasis effects corresponding to QTSs for cholesterol (Table 2). We reanalyzed cholesterol data by ignoring dominance and epistasis effects and observed substantial difference in analysis results as compared to the full model approach. For example, some new loci were identified, and some full-model detected loci were disappeared (Table S1, Fig. 1). Multi-loci additive model identified 5 new QTSs, but did not detect 4 QTSs compared to the full model approach. We observed no ethnic specific effects highly significant in this case. Two QTSs (rs7694118 and rs10768634) had only highly significant dominance effects detected by full model approach, and were not detectable by multi-loci additive model due to ignoring non-additive effects. However, two QTSs (rs6465748 and rs10483461) had highly significant additive and ethnic specific additive effect through full model, but also had not been detected through multi-loci additive model. Most probable reason is due to ignoring dominance and epistasis effects. In general, phenotypic traits follow joint or multivariate distributions. If, we ignore some important variants from genetic model and fit the remain variants with phenotype, the effects of ignoring factors could reduce detection power of loci. By analyzing cholesterol data through multi-loci additive model, we identified some QTSs that were not detected by full model. Probably, those QTSs were falsely detected due to ignoring dominance and epistasis effects or full model approach failed to detect them.

### Association study through *PLINK* and *GCTA*

We also analyzed cholesterol data using single locus approaches by adjusting for population stratifications and genetic relatedness through principle components and genetic relationship matrix (GRM). For *PLINK* analysis, sex and top 10 PCs were included in the analysis as covariates to account the potential variations due to population stratification and sex differences within Multi-Ethnic Study of Atherosclerosis (MESA) population. For *GCTA* analysis, GRM was additionally used to control genetic relatedness. We separately analyzed cholesterol data for two subsequent Examinations (Exam-1 and Exam-3) of MESA population. Genomic inflation factors (λ_GC_). which were calculated based on median of Chi-square distribution of whole-genome *P*-values, were 1.02 and 1.03 for Exam-1 and Exam-3 respectively after adjusting the population stratification by 10 principle components (PCs). Again, Q-Q plots of the GWASs *P*-values from *PLINK* and *GCTA* analyses showed good-fit to the uniform distribution (Figs S1B~S4B). Therefore, 10 PCs had efficiently controlled the population stratifications.

For *GCTA* analysis, we used MLMe^20^ approach that ignores the testing chromosome in time of constructing GRM and in addition the 10 PCs and sex were used as cofactors. We used linkage disequilibrium (LD) based Bonferroni corrected threshold to determine genome-wide significance for *PLINK* and *GCTA* analyses. By setting “--indep-pairwise 50 5 0.75” option in *PLINK* v1.07, we removed the SNPs of high LD (r^2^ > 0.75) with the extracted set of SNPs, and calculated the approximate number of the independent tests. After the LD based pruning, there were 458,716 SNPs and therefore Bonferroni corrected threshold for genome-wide significance at α = 0.05 level of significance was −log_10_*P* = 6.96.

We inspected additive and dominance effects using *PLINK*, however none of the dominance effects were genome-wide significant. Therefore, we recalculated the effects of all SNPs for additive effects only, and detected only two loci for Exam-1 and one locus for Exam-3, respectively with genome-wide significant for additive effects. Number of non-missing individual observations was larger in Exam-1 (*N* = 5277) as compared to Exam-3 (*N* = 4572), and therefore the power of SNP detection was better in Exam-1 as compared to Exam-3. The QTS rs629301 (−log_10_*P* = 12.10) located at chromosome-1 and the QTS rs478442 (−log_10_*P* = 6.97) located at chromosome-2 was genome-wide significant for Exam-1 (Table S2, Fig. S1A) and only the QTS rs629301 (−log_10_*P* = 8.37) was genome-wide significant for Exam-3 (Table S3, Fig. S2A). Analysis with *GCTA* detected only one significant locus, the QTS rs629301 with genome-wide significance (−log_10_*P* = 11.56 for Exam 1 and −log_10_*P* = 8.20 for Exam 3) for the both Exams (Table S2 and Table S3, Fig. S3A and Fig. S4A). Remarkably, the QTSs rs629301 and rs478442 were also detected by using full model and multi-loci additive model approach. Again, most of the candidate QTSs detected through full model approach had significant effects in *PLINK* and *GCTA* analyses for both Exams, although their effects were not genome-wide significant (Table S2 and Table S3). The significance of the candidate QTSs in both Exams might refer their robustness of associations with cholesterol data.

### Simulation studies

Under the simulated scenario-I, calculated powers of detecting loci using full model were high or moderate for most of the loci (Table S4). Again, calculated powers of detecting effects were high or moderate for most of the effects corresponding to loci (Table 3). Full model approach detected two loci (rs629301 and rs478442) with full power, nine loci with high power (>70%), five loci with moderate power (>40%) (Table S4). We observed detection powers were low for the effects of loci that were not highly significant in real data analysis using the full model approach. For example, estimated effects of the QTS rs7624679 were not highly significant in real data analysis and therefore in simulation study the detection powers of the effects were also low (9% for additive effect and only 2% for dominance by ethnic interaction effects). Again, QTS rs629301 had highly significant additive effect (−log_10_*P*_*EW*_ > 34) but low significant additive by ethnic interaction effect (−log_10_*P*_*EW*_ > 2.70), whereas detection powers were 100% for additive effect but only 15% for additive by ethnic interaction effect. Full model approach can obtain unbiased estimates of parameters and produce only small FDR (3.7%) under the simulated scenario-I.

We used multi-loci additive model approach ignoring dominance and epistasis effects to analyze the simulated traits. In this case, calculated FDR was equal to 15.5%, indicating that significantly increase of advocate FDR could be due to ignoring dominance and epistasis effects from reduced model. However, most of the detected false loci using full model and multi-loci additive model had very high LD (r^2^ > 0.85) with the true loci, might be representative of true loci. We observed most of the false loci were very near to the true loci and had similar effect size. If we consider the false loci as the representative of true loci, then false discovery rates were close to zero for the full model and multi-loci additive model. However, multi-loci additive model identified more nearby false loci as compared to the full model. Again, estimation of genetic parameters was biased upwards or downwards for some loci (Table 4). Total 13 loci were detected out of 17 true loci (4 loci were failed to detect), and none of the ethnic specific effects were significantly detected. None of the falsely identified loci had high LD with the four loci that were failed to detect in 100 simulations using multi-loci additive model.

It was suggested that additive model approach could result in biasedness or deficiency if dominance and epistasis effects have impacts on complex traits. By comparing the results from full model and multi-loci additive model in the contexts of real and simulated data analyses, we can reveal the possible explanations of differences in results from these two approaches for cholesterol data analysis. From simulation results, we observed that multi-loci additive model failed to detect QTSs rs6465748 and rs10483461 for 100 simulations, suggesting that estimates of the genetic effects of those QTSs did not reach to experimental-wise significance through additive model approach, although they had true additive effect. In real data analyses, we also observed those QTSs were not detected through additive model but detected by full model (Table 3). Therefore, ignoring important genetic factors could reduce power of detecting QTSs. QTSs with only dominance effects were not detected in simulated data analyses by multi-loci additive model. It was revealed that reduce model could not detect some true QTSs due to ignoring dominance and epistasis effects, and result in biased estimations and increase FDR.

**Figure 3:**
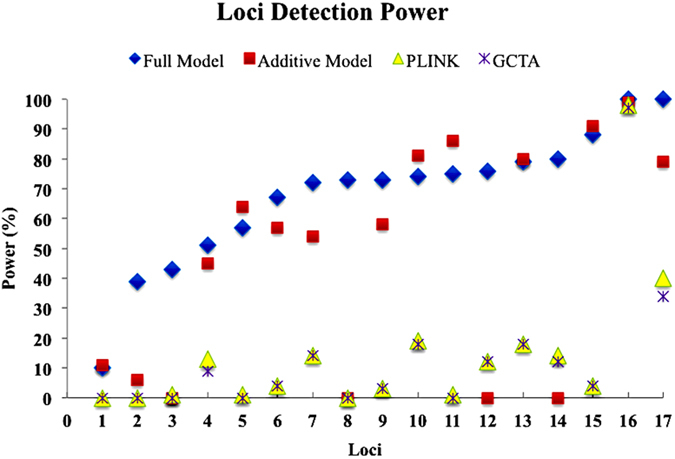
Powers of detecting loci of different mapping approaches. Loci were sorted according to detection power of full model approach.

For *PLINK* and *GCTA* analyses, calculated powers under the simulated scenario were high and moderate for two SNPs (rs629301 and rs478442) identified in real data analysis using single locus approaches (Tables S2~S4). Calculated powers for others SNPs were quite low and mostly not detectable using single locus approaches (Fig. 3). Therefore, the simulation results supported the real data analyses using different genetic model approaches, and revealed usefulness of the full model approach to analyze complex traits.

Comparisons between single-locus approaches revealed that detection powers of *PLINK* analysis were better than *GCTA* analysis (Table S4). Therefore, over controlling also can decrease power for detecting true QTSs. By comparing loci detection powers of different approaches, it was revealed that full model approach is more powerful to dissect genetic architecture of complex traits (Fig. 3).

Under scenario-II, we assume causal markers are controlling simulated traits in additive fashion with their additive and additive by ethnic interactions. In this case, both full model and multi-loci additive model could provide unbiased estimate of genetic parameters (Table S5). False discovery rate was also similar for these two approaches, 4.85% for full model approach and 3.96% for multi-loci additive model approach. Under this scenario, additive model identified all ethnic specific genetic effects that support our hypothesis (Table S5). Because, under this scenario additive model approach did not have extra noise rather than random errors in estimating parameters. Detection powers were similar for full model and multi-loci additive model approaches. Under this scenario-II, single locus approach of *PLINK* and *GCTA* could also suffer in decreasing detection power for most of the true loci (Table S6).

## Discussions

Genetic effects of multiple loci might differ in different human ethnicity due to various lifestyles and environmental exposures, and therefore analyzing gene by ethnicity interactions might help to reveal better knowledge about complex traits and personalization of treatment after a disease^44^,^45^. Our study provides underlying genetic mechanism of total cholesterol based on MESA population, and demonstrates how genetic effects of multiple loci might differ across the ethnic groups (gene by ethnicity interactions). Association mapping with full model and multi-loci additive genetic model includes gene by ethnicity interactions as random effects could estimate genetic effects of genes in specificities. In this study, full model approach estimated around 14% heritability due to ethnic specific effects that illustrate the importance of analyzing ethnic specific effects of genetic variants. Different genetic effects (e.g. additive, dominance, and epistasis) are also expected to have influence on complex traits^46^,^47^,^48^, however most of the association studies ignore dominance and epistasis effects^49^,^50^. Full model approach estimated around 11.24% heritability for dominance and epistasis effects and 10.21% heritability for ethnic specific dominance and epistasis effects. Multi-loci additive model approach ignored dominance and epistasis effects under assumption that only additive effects of multiple loci control the trait. However, ignoring dominance and epistasis effects might create biased in analysis results and cause missing heritability. In this study, total estimated heritability for cholesterol trait was 33.64% by using full genetic model approach, but was only 13.91% by using multi-loci additive model approach. Estimated heritability was 21.92% through *GCTA* for Exam-1 data, but only one SNP was detectable with genome-wide significance. It was suggested that there might have some other causal loci controlling the cholesterol trait of MESA population.

For full model and multi-loci additive model approaches, we used repeated measures from two-year examinations (Exam-1 and Exam-3) for subjects as replications to improve power of detecting small effects of multiple causal loci. Comparisons of full model and multi-loci additive model provide insight of usefulness of full model and the consequences of ignoring dominance and epistasis effects. In simulation study under two different scenarios, we observed that full model approach could obtain unbiased estimation of genetic parameters with only a small fraction of FDR (3.70~4.85%) with repeated measures. Again, under scenario-II, we observed that the full model and multi-loci additive model could obtain unbiased estimates of the genetic parameters with similar FDR, indicating robustness of the full model approach even only additive genetic effects of multiple loci controlling phenotypic traits. Multi-loci additive model provided biased estimates of several genetic parameters, failed to detect several true loci and ethnic specific genetic effects of loci in 100 simulations under the scenario-I, describing the deficiencies of the additive model approach for complex trait analysis.

Detection power of QTSs was very low for single locus approaches (*PLINK* and *GCTA*) under two different simulated scenarios. This result could describe the reasons of difference in results of single locus approaches from full model approach in our real data analyses. Separately analyses of data sets for Exams-1 and Exams-3 through *PLINK* and *GCTA* can detect only two different loci associated with cholesterol (Table S2 and Table S3). In simulation study, we observed that single locus approach detected QTS rs629301 with high power and QTS rs478442 with moderate power, but detection powers for other loci were negligible. Again, average detection power of multi-loci additive model approach was lower than the full genetic model approach. It was revealed that multi-loci additive model approach failed to detect some QTSs of cholesterol trait due to ignoring dominance and epistasis effects. Therefore, detection power of full model approach could be dramatically increased not only due to using repeated measures but also including dominance and epistasis effects into the model.

Association using full model approach identified the variants of three known genes *CELSR2, APOB* and *CETP* that were reported in previous association studies for cholesterol^4^,^25^. Additionally, the full model approach identified several new genes associated with cholesterol. Bioinformatics analysis revealed a complex networks through functions, protein-protein interactions or pathway interactions among the detected genes (Fig. 2). Disease-phenotype association study showed some newly detected genes associate with several cardiovascular diseases (Fig. 2). By searching functions of some newly detected genes, it seems that they have associations with cholesterol. For example, previous experimental analyses showed the gene *UFM1* increase the macrophage cholesterol efflux, which might due to the increased expression of ATP-binding cassette transporters A1 (*ABCA1*) and G1 (*ABCG1*)^51^; *ABCA1* gene was reported to be associate with total cholesterol in previous genome-wide association study (GWAS)^5^. Previous GWAS reported the 400 kb upstream variant of *UMF1* to be associate with plasma lipoprotein levels^52^. Highly significant epistasis effects between the variants of *SKAP2* and *UFM1* genes were detected for additive-by-additive and dominance related interactions in our study, where bioinformatics ontology search showed that they are related through cytoplasm. *ETFA* (electron transfer flavoprotein A) has functions for multiple acyl-coenzyme A dehydrogenation deficiency (MADD), and is related with lipid storage myopathies (LSMs)^53^. Zebrafish mutant dark Xavier (dxa^vu463^) in the *ETFA* gene has swollen and hyperplastic neural progenitor cells, hepatocytes, and kidney tubule cell as well as elevations in triacylglycerol, cerebroside sulfate and cholesterol levels^54^. Therefore, detected genes through full model approach might be biologically plausible for cholesterol trait.

## Methods

### Data

Multi-Ethnic Study of Atherosclerosis (MESA) data used in this study were downloaded from dbGaP (database of Genotypes and Phenotypes, http://www.ncbi.nlm.nih.gov/gap). MESA is a prospective population-based study focusing on characterization of subclinical cardiovascular disease and the risk factors that enable prediction of the progression of CVD55. Study participants of four ethnic groups include 6,500 men and women, nearly in equal numbers, who are aged 45~84 years and free of clinical CVD at baseline, and initially recruited in 2000 from six US communities: Baltimore, MD; Chicago, IL, Forsyth County, NC; Los Angeles County, CA, Northern Manhattan, NY; and St. Paul, MN. The recruited participants are 38% European-American (E-A), 28% African-American (A-A), 22% Hispanic American (H-A), and 12% Asian, predominantly of Chinese descent, American (C-A). More details of sampling design and study procedures have described previously by Bild *et al*.^55^. We excluded SNPs with MAF < 0.05, call rate < 90% for analysis by using PLINK-v1.07. We did not test for Hardy-Weinberg equilibrium due to heterogeneity structure of MESA population (Fig. S5A). After applying the above QC filtering criteria, a total of 714,211 SNPs were included in analysis. LD based SNP pruning was done only for calculating approximate number of independent test to set genome-wide significance threshold of single locus mapping approach. Genotype clusters of ethnic groups were observed using 3D scatter plot based on first 3 Eigen vectors (Fig. S5A).

Phenotype data from two different examinations (Exam-1: July 2000–July 2002; and Exam-3: January 2004–July 2005) were used in this study^55^. We observed the random heterogeneity among the distributions of cholesterol according to ethnic groups (Fig. S5B). Again, significant sex differences observed within each ethnic group. Therefore, sex was used as block and ethnic effects were used as random factors to control confounding due to sex and ethnic effects in our analyses. Cholesterol data for MESA population were analyzed using *QTXNetwork* and checked for outliers using residuals of the model. For full model and multi-loci additive model approaches, we discarded phenotypic outliers using standardized residual analysis, and reanalyzed data.

### Statistical Analyses

Two distinct approaches were used for genome-wide association analyses of cholesterol trait: generalized multi-factor dimensionality reduction (GMDR) method to scan 714,211 SNPs by 1D for main effects, 2D and 3D for epistasis interactions using module *GMDR*-GPU^56^ in *QTXNetwork.* And then association mapping was conducted by *QTS* module of *QTXNetwork* for detected 707 candidate SNPs. The linear mixed model includes SNP loci effects (*a, d, aa, ad, da, dd*) as fixed; ethnicity (*e*) and loci by ethnicity interaction (*ae, de, aae, ade, dae, dde*) as random effects,


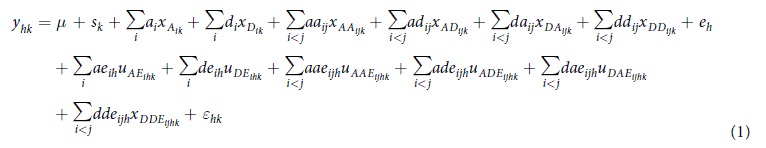


where μ is the population mean; *s*_*k*_ is the fixed effect of the *k*-th individual (0 for female, 1 for male); *a*_*i*_ is the additive effect of the *i*-th locus with coefficient 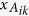 (1 for *QQ*, 0 for *Qq*, −1 for *qq*); *d*_i_ is the dominance effect of the *i*-th locus with coefficient 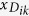 (1 for *Qq,* 0 for *QQ* and *qq*); *aa*_*ij*_, *ad*_*ij*_, *da*_*ij*_ and *dd*_*ij*_ are the digenic epistasis effects with coefficients 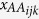 (1 for *QQ* × *QQ* and *qq* × *qq,* −1 for *QQ* × *qq* and *qq* × *QQ*, and 0 for others), 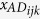 (1 for *QQ* × *Qq,* −1 for *qq* × *Qq*, and 0 for others), 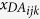 (1 for *Qq* × *QQ,* −1 for *Qq* × *qq*, and 0 for others) and 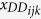 (1 for *Qq*  × *Qq*, and 0 for others); *e*_*h*_ is the effect of the *h*-th ethnic population (1 for *E-A*, 2 for *C-A*, 3 for *A-A*, 4 for *H-A*); *ae*_*ih*_ is the additive × race interaction effect of the *i*-th locus in the *h*-th ethnic population with coefficient 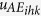; *de*_*ih*_ is the dominance × race interaction effect of the *i*-th locus in the *h*-th ethnic population with coefficient 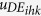; *aae*_*ijh*_, *ade*_*ijh*_, *dae*_*ijh*_ and *dde*_*ijh*_ are the digenic epistasis × race interaction effects in the *h*-th ethnic population with coefficient 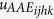, 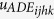, 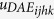 and 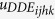; and *ε*_*hk*_ is the residual effect of the *k*-th individual in the *h*-th ethnic population. In this model, we have constraints for random variables with normal distributions of zero mean and variances 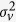.

The linear mixed model and its distribution can be expressed in matrix notation,


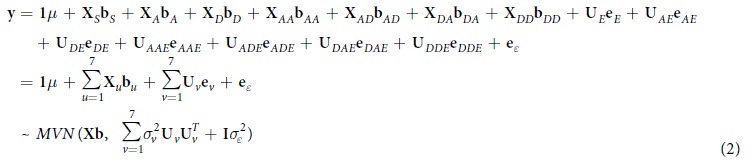


where **y** is an *n* × 1 column vector of phenotypic values and *n* is the sample size of observations; *μ* is the population mean, **b**_*u*_ is the *u*-*th* vector of fixed effects; **X**_*u*_ is the known incidence matrix relating to the *u*-*th* fixed effects; **e**_*v*_ is the *v*-*th* vector of random effects with distribution 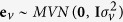; **U**_*v*_ is the known coefficient matrix for the *v*-*th* vector of random effects; 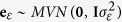 is an column vector of residual effects.

The total heritability is estimated by


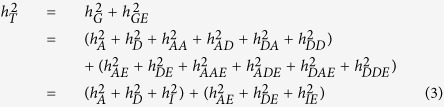


where 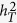 is the total heritability; 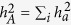 is the heritability due to additive effects contributed by sum of individual locus, 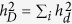 is the heritability due to dominance effects contributed by sum of individual locus, 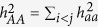 is the heritability contributed by sum of pair-wise additive by additive (*aa*) epistasis, 
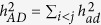
 is the heritability contributed by sum of pair-wise additive by dominance (*ad*) epistasis, 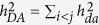 is the heritability contributed by sum of pair-wise dominance by additive (*da*) epistasis, 
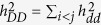
 is the heritability contributed by sum of pair-wise dominance by dominance (*dd*) epistasis, 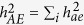 is additive by environment interaction heritability contributed by sum of individual additive by environment interaction effects, 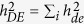 is dominance by environment interaction heritability contributed by sum of individual dominance by environment interaction effects, 
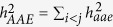
 is *aa* epistasis by environment interaction heritability contributed by sum of pair-wise *aa* epistasis by environment interaction effects, 
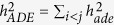
 is *ad* epistasis by environment interaction heritability contributed by sum of pair-wise *ad* epistasis by environment interaction effects, 
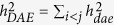
 is *da* epistasis by environment interaction heritability contributed by sum of pair-wise *da* epistasis by environment interaction effects, 
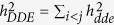
 is *dd* epistasis by environment interaction heritability contributed by sum of pair-wise *dd* epistasis by environment interaction effects.

We also used a multi-loci additive model by ignoring non-additive effects in our study with the following form


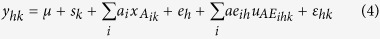


with matrix notation and distribution as


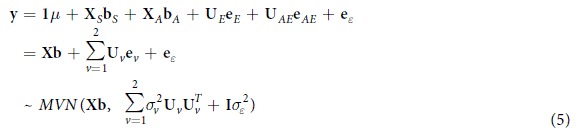


The heritability of a multi-loci additive model is estimated by


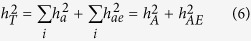


The linear mixed model with Henderson method III^57^ was used to construct the *F*-statistic test for association analysis. Permutation test was conducted by a total of 2,000 times for calculating the critical *F*-value to control the experiment-wise type I error (*α*_*EW*_ < 0.05). The QTS effects were estimated by using the MCMC (Markov Chain Monte Carlo) algorithm with 20,000 Gibbs sample iterations^6^,^22^,^58^,^59^. The critical experiment-wise *P* value (*P*_EW_-value) for genetic effects by controlling the experiment-wise type I error (*P*_EW_ < 0.05) was thus calculated. More details of procedure about the approaches were described in S1 text.

### Simulation Design

Association analyses of cholesterol data by using full model and additive models revealed different results. We conducted Monte-Carlo simulations to perceive possible explanations of differences in results using full model and additive models. Simulated phenotypic data were generated under two distinctive scenarios: (I) simulated traits controlling by additive, non-additive genetic effects and ethnic specific genetic effects; (II) simulated traits controlling by additive and their ethnic interaction effects. We generated data for repeated measures of individuals and analyzed by treating as replications through full model and multi-loci additive model approaches. Under scenario-I, estimated genetic effects of significant loci obtained from full model approach were used as true loci and parameters to generate phenotypic data, and estimated error variance used to generate random errors. In Table 2, we presented only outcomes for the genetic effects of QTSs that were experimental-wise highly significant (*P*_*EW*_≤ 1 × 10^−5^) through full model approach. However, this approach also detected some experimental-wise significant effects with *P*_*EW*_ < 0.05. In simulation study, we used all perceived effects of loci; because small effects of several variants also might have influence on complex traits. Under scenario-II, we simulated data in similar way as scenario-I nevertheless using perceived genetic architecture of cholesterol trait from multi-loci additive model approach. Therefore, loci truly had only additive and additive-by-ethnic interaction effects in scenario-II.

For association analyses through full model and multi-loci additive model approaches, false positive rates (FDR) were estimated as the ratio of falsely identified loci with respect to the total number of detected loci for each simulated trait, detection powers of loci were the detection rate of each locus, and detection power of the effects were the detection rate of the effects in 100 simulations. Powers of single-locus additive model approaches were estimated in similar way. We estimated empirical confidence interval of each effects from output of 100 simulations, and described estimation is unbiased if parameter belonging to the empirical confidence interval otherwise estimation is biased. We did not arrange empirical confidence intervals in table, however biased estimates were marked by “^+^” or “^−^” sign at the right side of estimated value, whereas “^+^” refers overestimation and “^−^” refers underestimation of parameters (Table 3 and Table 4).

We also analyzed the simulated data by using single locus approaches implemented in *PLINK* and *GCTA*. Only detection power of loci were presented for *PLINK* and *GCTA* analyses (Fig. 3, Table S4), because their estimates of parameters can be different from true value of parameters due to different coding system for genotype data. Again, repeated measures of subjects could not be used by these two approaches, because the software can not analyze data with replications. We define SNPs as detected while its calculated *P*-value exceeds LD-based Bonferroni corrected threshold.

## Additional Information

**How to cite this article**: Monir, M. M. and Zhu, J. Comparing GWAS Results of Complex Traits Using Full Genetic Model and Additive Models for Revealing Genetic Architecture. *Sci. Rep.* **7**, 38600; doi: 10.1038/srep38600 (2017).

**Publisher's note:** Springer Nature remains neutral with regard to jurisdictional claims in published maps and institutional affiliations.

## Supplementary Material

Supplementary Information

## Figures and Tables

**Table 1:** Estimated heritability of cholesterol using full and multi-loci additive model.

Model	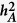 (%)	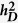 (%)	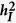 (%)	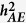 (%)	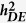 (%)	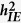 (%)	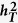 (%)
Full	8.06	2.67	8.57	4.13	6.54	3.67	33.64
Additive	10.55			3.36			13.91

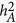
 = additive heritability, 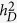 = dominance heritability, 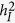 = epistasis heritability, 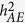 = additive by ethnic heritability, 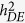 = dominance by ethnic heritability, 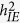 = epistasis by ethnic heritability, 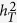 = total heritability.

**Table 2:** Identified QTSs for cholesterol using full model approach.

Chr_SNP_Alleles	Gene	Effect	Estimate	SE	−log_10_*P*_*EW*_	*h*^2^ (%)
1_rs629301_T/G	*CELSR2*	*a*	4.94	0.40	34.47	1.46
2_rs478442_A/C	132 kb 5′ of *APOB*	*a*	5.73	0.39	47.61	1.97
		*de*_*1*_	−3.85	0.85	5.23	1.32
		*de*_*4*_	5.40	1.26	4.75	
4_rs7694118_C/T	1.8 kb 5′ of *PCDH10*	*d*	3.11	0.64	6.01	0.58
		*de*_*1*_	8.31	2.12	5.04	3.18
		*de*_*3*_	−6.08	0.96	9.60	
5_rs17409624_G/A	*DROSHA*	*a*	2.14	0.45	5.70	0.27
7_rs6465748_G/A	9.6 kb 5′ of *MYH16*	*a*	−3.11	0.35	18.43	0.58
		*ae*_*3*_	−4.67	0.68	11.12	1.22
		*ae*_*4*_	4.33	0.71	9.04	
8_rs10503377_C/T	139 kb 5′ of *SGK223*	*a*	3.16	0.42	13.50	0.60
11_rs10768634_T/C	*OR52A1*	*d*	−3.45	0.64	7.19	0.71
11_rs7124873_G/A	10 kb 3′ of *MMP13*	*a*	2.57	0.44	8.13	0.40
14_rs10483461_C/T	117 kb 3′ of *BRMS1L*	*a*	2.35	0.34	11.05	0.33
		*ae*_*3*_	4.02	0.69	8.17	0.97
15_rs12595211_G/A	*ETFA*	*a*	−2.52	0.42	8.51	0.38
16_rs1532625_G/A	*CETP*	*a*	−2.85	0.43	10.46	0.49
17_rs2649485_C/T	3.3 kb 5′ of *CCDC144B*	*a*	−2.58	0.45	8.12	0.40
19_rs1654452_C/T	*RDH13*	*a*	−2.02	0.43	5.64	0.25
2_rs478442_A/C ×	132 kb 5′ of *APOB* ×	*aa*	2.38	0.46	6.77	0.34
4_rs7694118_C/T	1.8 kb 5′ of *PCDH10*	*da*	3.59	0.67	7.09	0.77
7_rs2264802_C/A ×	*SKAP2* × *UFM1*	*aa*	−4.71	0.44	26.41	1.33
13_rs9548318_G/A		*ad*	−6.14	0.86	12.16	2.26
10_rs12246594_A/T ×	*SORCS1* × *ETFA*	*aa*	7.46	0.44	13.34	3.34
15_rs12595211_G/A						

Chr_SNP_Alleles: chromosome_SNP_major/minor alleles; Gene: the near or holder genes corresponding to QTSs; Effect: genetic effects of QTSs, *a* = additive effect, *d* = dominance effect, *aa* = additive-additive epistasis effect, *ad* = additive-dominance epistasis effect, *da* = dominance-additive epistasis effect, *ae*_*3*_ = *A-A* specific additive effect, *ae*_*4*_ = *H*-*A* specific additive effect, *de*_*1*_ = *E-A* specific dominance effect, *de*_*3*_ = *A-A* specific dominance effect, *de*_*4*_ = *H-A* specific dominance effect; Estimate: the estimated genetic effects; −log_10_*P*_*EW*_: minus log_10_ (experimental-wise *P*-value); *h*^*2*^ (%) refers heritability in percentage.

**Table 3:** Monte-Carlo simulation study of full model approach.

Chr_SNP_Alleles	Effect	Parameter	Estimate	SE	Power (%)
1_rs629301_T/G	*a*	4.94	5.80	0.80	100
	*ae*_*4*_	1.75	1.90	0.29	15
1_rs2499595_G/A	*a*	1.86	1.96	0.52	44
	*ae*_*1*_	1.36	1.90	0.38	23
	*ae*_*2*_	−3.73	−3.20	0.57	37
	*ae*_*4*_	1.94	2.43	0.51	20
2_rs478442_A/C	*a*	5.73	5.19	0.53	81
	*ae*_*1*_	−2.37	−0.46	2.93	7
	*de*_*1*_	−3.85	0.58	3.82	14
	*de*_*3*_	−2.91	−3.83	2.04	42
	*de*_*4*_	5.40	3.84	1.88	27
3_rs7624679_C/T	*a*	1.47	2.31	0.40	9
	*de*_*4*_	−1.81	−3.54	1.97	2
4_rs7694118_C/T	*d*	3.11	2.56	1.38	50
	*de*_*1*_	8.31	6.38	1.61	51
	*de*_*3*_	−6.08	−3.81	1.94	51
5_rs17409624_G/A	*a*	2.14	2.31	0.47	78
	*ae*_*3*_	3.14	2.99	0.85	71
	*ae*_*4*_	−3.00	−2.95	0.73	69
7_rs6465748_G/A	*a*	−3.11	−2.85	2.15	37
	*d*	2.14	4.41	1.58	29
	*ae*_*1*_	1.55	1.26	1.72	20
	*ae*_*3*_	−4.67	−5.92	2.43	35
	*ae*_*4*_	4.33	4.51	1.75	34
8_rs10503377_C/T	*a*	3.16	3.16	0.75	73
	*ae*_*1*_	−2.12	−2.29	0.68	61
	*ae*_*2*_	4.74	4.41	1.32	69
	*ae*_*4*_	−2.30	−2.59	0.57	44
11_rs10768634_T/C	*a*	−1.15	0.72	1.05	21
	*d*	−3.45	−3.93	0.82	55
11_rs7124873_G/A	*a*	2.57	2.97	0.42	76
14_rs10483461_C/T	*a*	2.35	2.86	2.03	67
	*d*	−2.73	−4.50	1.68	60
	*ae*_*3*_	4.02	2.96	1.08	23
	*ae*_*4*_	−1.47	−2.23	1.28	16
15_rs12595211_G/A	*a*	−2.52	−2.98	0.58	51
	*de*_*4*_	1.94	2.45	0.63	15
16_rs1532625_G/A	*a*	−2.85	−3.19	0.65	79
16_rs4888652_T/C	*a*	1.91	2.43	0.46	46
17_rs2649485_C/T	*a*	−2.58	−2.84	0.39	64
18_rs9946067_T/C	*a*	−2.23	−1.63	0.46	36
	*d*	2.89	2.70	0.77	39
19_rs1654452_C/T	*a*	−2.02	−2.29	0.53	70
	*d*	1.62	2.00	0.64	64
2_rs478442_A/C ×	*aa*	2.38	2.83	0.69	12
4_rs7694118_C/T	*ad*	−2.60	−4.14	1.69	10
	*da*	3.59	−4.14	1.69	11
16_rs4888652_T/C ×	*da*	2.64	−0.58	1.74	10
18_rs9946067_T/C	*dd*	−1.95	−3.52	0.95	8
7_rs2264802_C/A ×	*aa*	−4.71	−4.21	0.85	10
13_rs9548318_G/A	*ad*	−6.14	−6.45	0.75	8
10_rs12246594_A/T×	*aa*	7.46	6.86	1.08	12
15_rs12595211_G/A					

Results of Monte-Carlo simulations under scenario-I. Chr_SNP_Alleles: chromosome_QTS_major/minor alleles; Effect: mode of gene action; Parameter: true genetic effect; Estimate: average estimated effect of genetic parameters; Power (%): power of detecting effects in percentage.

**Table 4:** Monte-Carlo simulation study of multi-loci additive model.

Chr_SNP_Alleles	Effect	Parameter	Estimate	SE	Power (%)
1_rs629301_T/G	*a*	4.94	5.81	0.57	99
1_rs2499595_G/A	*a*	1.86	1.89	0.49	57
2_rs478442_A/C	*a*	5.73	3.81^**−**^	0.52	79
3_rs7624679_C/T	*a*	1.47	2.46^**+**^	0.42	11
5_rs17409624_G/A	*a*	2.14	2.20	0.49	91
8_rs10503377_C/T	*a*	3.16	3.33	0.43	58
11_rs10768634_T/C	*a*	−1.15	2.93^**+**^	0.42	86
15_rs12595211_G/A	*a*	−2.52	−3.09	0.50	45
16_rs1532625_G/A	*a*	−2.85	−3.25	0.48	80
16_rs4888652_T/C	*a*	1.91	2.24	0.40	64
17_rs2649485_C/T	*a*	−2.58	−2.84	0.39	81
18_rs9946067_T/C	*a*	−2.23	−2.75^−^	0.30	6
19_rs1654452_C/T	*a*	−2.02	−3.31	0.38	54

Results of Monte-Carlo simulation under scenario-I. Chr_SNP_Alleles: chromosome_QTS_major/minor alleles; Estimate: average estimated effects of genetic parameters; SE: standard errors; Power (%): power of detecting QTSs in percentage; “+” and “−” signs at the right side of the estimated value refers overestimate and underestimates corresponding to the parameters.
