# Supplementary material for: Comparing GWAS Results of Complex Traits Using Full Genetic Model and Additive Models for Revealing Genetic Architecture
Source: Sci Rep. 2017 Jan 12;7:38600. doi: 10.1038/srep38600 (PMC5227710; doi:10.1038/srep38600)
Supplement: Supplementary Information [file srep38600-s1.doc]

**­­­Comparing GWAS Results of Complex Traits Using Full Genetic Model and Additive Models for Revealing Genetic Architecture**

Md. Mamun Monir and Jun Zhu*

Institute of Bioinformatics, Zhejiang University, Hangzhou 310058, China

*Corresponding author

Email: [jzhu@zju.edu.cn](mailto:jzhu@zju.edu.cn)

**S1 text. Statistical Methods of QTS module**

In the section of materials and methods, we briefly described the statistical approaches used in this study. We presented the detailed process of *QTXNetwork* as follows,

**Individual locus detection**. Significance testing was performed for each individual SNP locus in this step. In full model approach, to test the significance of the *i*-th individual SNP locus, we used the following linear mixed model,

$y_{hk}=\mu+s_{k}+a_{i}x_{A_{ik}}+d_{i}x_{D_{ik}}+e_{h}+ae_{ih}u_{AE_{ihk}}+de_{ih}u_{DE_{ihk}}+\varepsilon_{hk}$ (1)

where, the definitions of parameters and coefficients are the same as we defined in the materials and methods section of the main text.

In the case of multi-loci additive model, we used the following linear mixed model,

$y_{hk}=\mu+s_{k}+a_{i}x_{A_{ik}}+e_{h}+ae_{ih}u_{AE_{ihk}}+\varepsilon_{hk}$ (2)

where, notations of the equation-2 are similar as defined above. Based on the model defined in equation 1 and 2, the *F*-test based on Henderson method III was used for significance testing for full model and multi-loci additive model respectively. The locus with *F*-value exceeding the predefined threshold value is considered as a candidate individual SNP. To determine threshold value of the statistic, permutation test was conducted by a total of 2,000 times for calculating the critical *F*-value to control the experiment-wise type I error (< 0.05).

**Epistasis loci detection**. Given on the effects of the *m* significant candidates selected by step 1, a two-way interaction significance testing for all possible SNP pairs was performed. The model for testing the significance of epistasis interaction between the *i*-th SNP locus and *j*-th SNP locus can be written as

$\begin{matrix} y_{hk}=\mu+s_{k}+\sum_{i}^{m} (a_{i}x_{A_{ik}}+d_{i}x_{A_{ik}})+ aa_{ij}x_{AA_{ijk}}+ad_{ij}x_{AD_{ijk}}+da_{ij}x_{DA_{ijk}}+dd_{ij}x_{DD_{ijk}} \\ \\ +e_{h}+\sum_{i}^{m} (ae_{ih}u_{AE_{ihk}}+de_{ih}u_{DE_{ihk}})+aae_{ijh}u_{AAE_{ijhk}}+ade_{ijh}u_{ADE_{ijhk}}+dae_{ijh}u_{DAE_{ijhk}}+dde_{ijh}u_{DDE_{ijhk}}+\varepsilon_{hk} \end{matrix}$ (3)

where, definition of the all parameters and coefficients were the same as their definition in materials and methods sections of the main text.

For multi-loci additive model epistasis searching model was the following

$y_{hk}=\mu+s_{k}+\sum_{i}^{m} a_{i}x_{A_{ik}}+e_{h}+ \sum_{i}^{m} ae_{ih}u_{AE_{ihk}}+aa_{ij}x_{AA_{ijk}}+aae_{ijh}u_{AAE_{ijhk}}+\varepsilon_{hk}$ (4)

Based on the mixed epistasis models, the *F*-test was performed to test all possible SNP pairs. The pair of loci with *F*-value larger than the predefined threshold value is considered as candidate epistasis interacting loci. To determine threshold value, permutation test was conducted by a total of 2,000 times for calculating the critical *F*-value to control the experiment-wise type I error (< 0.05).

**Model selection**. In this step, the stepwise model selections were performed on all previously significantly detected individual and pair-wise epistasis loci. Therefore, we obtained an optimum set of candidate individual and pair-wise loci for the full genetic model,

$\begin{matrix} y_{hk}=\mu+s_{k}+\sum_{i} a_{i}x_{A_{ik}}+\sum_{i} d_{i}x_{D_{ik}}+\sum_{i<j} aa_{ij}x_{AA_{ijk}}+\sum_{i<j} ad_{ij}x_{AD_{ijk}}+\sum_{i<j} da_{ij}x_{DA_{ijk}}+\sum_{i<j} dd_{ij}x_{DD_{ijk}} \\ +e_{h}+\sum_{i} ae_{ih}u_{AE_{ihk}}+\sum_{i} de_{ih}u_{DE_{ihk}}+\sum_{i<j} aae_{ijh}u_{AAE_{ijhk}}+\sum_{i<j} ade_{ijh}u_{ADE_{ijhk}}+\sum_{i<j} dae_{ijh}u_{DAE_{ijhk}}+\sum_{i<j} dde_{ijh}u_{DDE_{ijhk}}+\varepsilon_{hk} \end{matrix}$ (5)

And, for multi-loci additive model

$y_{hk}=\mu+s_{k}+\sum_{i} a_{i}x_{A_{ik}}+e_{h}+\sum_{i} ae_{ih}u_{AE_{ihk}}+\varepsilon_{hk}$ (6)

A matrix form for the above mixed linear model equations can be expressed as,

(7)

where, $\mathbf{y}\sim MVN(\mathbf{Xb}, \sum\sigma_{v}^{2}\mathbf{U}_{v}\mathbf{U}_{v}^{T}+\mathbf{I}\sigma_{\varepsilon}^{2})$ is an n × 1 column vector of phenotypic values and n is the sample size of observations; $\mathbf{b}_{u}$is the u-th vector of fixed effects with known incidence matrix $\mathbf{X}_{u}$ ; $\mathbf{U}_{v}$ is the known coefficient matrix relating to the *v*-th random vector $\mathbf{e}_{v}$ ; $e_{v}\sim MVN(0,I\sigma_{v}^{2})$ ; $\mathbf{e}_{\varepsilon}\sim MVN(\mathbf{0}, \mathbf{I}\sigma_{\varepsilon}^{2})$ is an n × 1 column vector of residual effects.

All parameters of the model were estimated by using MCMC (Markov Chain Monte Carlo) algorithm with 20,000 Gibbs sample iterations.

**Table S1. Results from Multi-loci Additive Model using QTS modular** of QTXNetwork

| Chr_SNP_Alleles | Gene | Effect | Predict | SE | **-**log_10_*P_EW_* | *h^2^*(%) |
| --- | --- | --- | --- | --- | --- | --- |
| 1_rs629301_T/G | *CELSR2* | *a* | 4.70 | 0.40 | 31.18 | 1.74 |
| 2_rs478442_A/C | 132kb 5' of *APOB* | *a* | 4.02 | 0.39 | 23.91 | 1.27 |
| 2_rs5743063_G/A | *PMS1* | *a* | -3.24 | 0.41 | 14.29 | 0.82 |
| 5_rs17409624_G/A | *DROSHA* | *a* | 2.08 | 0.45 | 5.44 | 0.34 |
| 6_rs2609326_C/T | *SMOC2* | *a* | 2.16 | 0.45 | 5.91 | 0.36 |
| 8_rs10503377_C/T | 139kb 5' of SGK223 | *a* | 3.04 | 0.42 | 12.53 | 0.73 |
| 11_rs7124873_G/A | 10kb 3' of *MMP13* | *a* | 2.75 | 0.45 | 9.14 | 0.59 |
| 13_rs1410165_G/A | 104kb 3' of *LOC338864* | *a* | 2.45 | 0.45 | 7.20 | 0.47 |
| 15_rs12595211_G/A | *ETFA* | *a* | -2.76 | 0.43 | 10.02 | 0.60 |
| 16_rs1532625_G/A | *CETP* | *a* | -3.10 | 0.43 | 12.17 | 0.75 |
| 16_rs4888652_T/C | *RP11-571O6.1* | *a* | 2.34 | 0.45 | 6.83 | 0.43 |
| 17_rs2649485_C/T | 3.3kb 5' of CCDC144B | *a* | -2.58 | 0.45 | 8.12 | 0.53 |
| 18_rs1105654_A/G | *-* | *a* | 2.32 | 0.43 | 7.14 | 0.42 |
| 19_rs1654452_C/T | *RDH13* | *a* | -2.84 | 0.43 | 10.44 | 0.63 |

Chr_SNP_Alleles: chromosome_QTS_major/minor alleles; Gene: the near or holder genes corresponding to QTSs; Effect: genetic effects of QTSs, *a* = additive effect; Predict: the predicted genetic effects; **-**log_10_*P_EW_*: minus log_10_ (experimental-wise P-value); *h^2^* (%) refers heritability in percentage.

**Table S2.** **Detected QTSs by using PLINK and GCTA for Examination 1**

| Chr_SNP_Alleles | Gene | PLINK | | | GCTA | | |
| --- | --- | --- | --- | --- | --- | --- | --- |
|  |  | Estimate | STAT | -log_10_*P* | Estimate | SE | -log_10_*P* |
| 1_rs629301_T/G | *CELSR2* | -5.56^a^ | -7.18 | 12.10 | -5.43**^a^** | 0.78 | 11.56 |
| 2_rs478442_A/C | 132kb 5' of *APOB* | -4.35^a^ | -5.32 | 6.97 | -4.28 | 0.84 | 6.47 |
| 4_rs7694118_C/T | 1.8kb 5' of *PCDH10* | -2.33 | -2.66 | 2.10 | -2.48 | 0.89 | 2.26 |
| 5_rs17409624_G/A | *DROSHA* | -2.60 | -3.99 | 4.17 | -2.40 | 0.66 | 3.52 |
| 7_rs6465748_G/A | 9.6kb 5' of *MYH16* | 5.22 | 4.08 | 4.34 | 5.07 | 1.29 | 4.24 |
| 8_rs10503377_C/T | 139kb 5' of *SGK223* | -2.83 | -3.85 | 3.92 | -2.69 | 0.76 | 3.70 |
| 11_rs7124873_G/A | 10kb 3' of *MMP13* | -2.24 | -3.37 | 3.12 | -2.24 | 0.68 | 3.15 |
| 14_rs10483461_C/T | 117kb 3' of *BRMS1L* | -5.05 | -3.74 | 3.73 | -4.70 | 1.37 | 3.40 |
| 15_rs12595211_G/A | *ETFA* | 2.79 | 4.01 | 4.22 | 2.79 | 0.71 | 4.07 |
| 16_rs1532625_G/A | *CETP* | 3.57 | 5.06 | 6.35 | 3.50 | 0.71 | 6.25 |
| 17_rs2649485_C/T | 3.3kb 5' of *CCDC144B* | 2.24 | 3.43 | 3.22 | 2.20 | 0.67 | 3.15 |
| 19_rs1654452_C/T | *RDH13* | 2.85 | 4.12 | 4.42 | 3.22 | 0.70 | 5.33 |

Chr_SNP_Alleles: chromosome_QTS_major/minor alleles; Gene: the near or holder genes corresponding to QTSs; Estimate: estimated additive effects; STAT: value of test statistic; SE: standard error; -log_10_P: -log_10_*P*-value. All of the tabulated SNPs had identified by using Full model approach.

a. Genome-wide significant effect of the corresponding SNP.

**Table S3.** **Detected QTSs by using PLINK and GCTA for Examination 3**

| Chr_SNP_Alleles | Gene | PLINK | | | GCTA | | |
| --- | --- | --- | --- | --- | --- | --- | --- |
|  |  | Estimate | STAT | -log_10_*P* | Estimate | SE | -log_10_*P* |
| 1_rs629301_T/G | *CELSR2* | -4.98^a^ | -5.89 | 8.37 | -4.93^a^ | 0.85 | 8.20 |
| 2_rs478442_A/C | 132kb 5' of *APOB* | -4.56 | -5.12 | 6.50 | -4.52 | 0.90 | 6.33 |
| 4_rs7694118_C/T | 1.8kb 5' of *PCDH10* | -1.99 | -2.05 | 1.39 | -1.96 | 0.97 | 1.37 |
| 5_rs17409624_G/A | *DROSHA* | -1.67 | -2.34 | 1.71 | -1.67 | 0.71 | 1.72 |
| 7_rs6465748_G/A | 9.6kb 5' of *MYH16* | 2.52 | 1.80 | 1.15 | 2.29 | 1.39 | 1.00 |
| 8_rs10503377_C/T | 139kb 5' of *SGK223* | -1.54 | -1.91 | 1.25 | -1.51 | 0.81 | 1.20 |
| 11_rs7124873_G/A | 10kb 3' of *MMP13* | -2.88 | -3.98 | 4.15 | -2.90 | 0.73 | 4.16 |
| 14_rs10483461_C/T | 117kb 3' of *BRMS1L* | -5.51 | -3.77 | 3.79 | -5.28 | 1.45 | 3.55 |
| 15_rs12595211_G/A | *ETFA* | 2.84 | 3.76 | 3.76 | 2.79 | 0.76 | 3.64 |
| 16_rs1532625_G/A | *CETP* | 2.66 | 3.46 | 3.26 | 2.62 | 0.77 | 3.21 |
| 17_rs2649485_C/T | 3.3kb 5' of *CCDC144B* | 3.21 | 4.49 | 5.14 | 3.13 | 0.72 | 4.88 |
| 19_rs1654452_C/T | *RDH13* | 2.94 | 3.88 | 3.98 | 2.84 | 0.76 | 3.75 |

Chr_SNP_Alleles: chromosome_QTS_major/minor alleles; Estimate: estimated additive effects; STAT: value of test statistic; SE: standard error; -log_10_P: -log_10_*P*-value. All of the tabulated SNPs had identified by using Full model approach.

a. Genome-wide significant effect of the corresponding SNP.

**Table S4. The power of detected loci by different** approaches under scenario-I

| Chr_SNP_Alleles | Full Model | Additive Model | PLINK | GCTA |
| --- | --- | --- | --- | --- |
| 1_rs629301_T/G | 100 | 99 | 98 | 97 |
| 1_rs2499595_G/A | 67 | 57 | 4 | 4 |
| 2_rs478442_A/C | 100 | 79 | 40 | 34 |
| 3_rs7624679_C/T | 10 | 11 | 0 | 0 |
| 4_rs7694118_C/T | 73 | 0 | 0 | 0 |
| 5_rs17409624_G/A | 88 | 91 | 4 | 4 |
| 7_rs6465748_G/A | 43 | 0 | 1 | 0 |
| 8_rs10503377_C/T | 73 | 58 | 3 | 3 |
| 11_rs10768634_T/C | 75 | 86 | 1 | 0 |
| 11_rs7124873_G/A | 76 | 0 | 12 | 12 |
| 14_rs10483461_C/T | 80 | 0 | 14 | 12 |
| 15_rs12595211_G/A | 51 | 45 | 13 | 9 |
| 16_rs1532625_G/A | 79 | 80 | 18 | 18 |
| 16_rs4888652_T/C | 57 | 64 | 1 | 0 |
| 17_rs2649485_C/T | 74 | 81 | 19 | 18 |
| 18_rs9946067_T/C | 39 | 6 | 0 | 0 |
| 19_rs1654452_C/T | 72 | 54 | 14 | 14 |

Chr_SNP_Alleles: chromosome_QTS_major/minor alleles; Full Model: Loci detection powers of full model, Additive Model: Loci detection powers of multi-loci additive model, PLINK: Loci detection powers of *PLINK* analysis, and GCTA: Loci detection powers of *GCTA* analysis.

**Table S5. Estimated effects and detected power of full model and multi-loci additive model under scenario-II.**

| **Chr_SNP_Alleles** | **Effect** | **Parameter** | **Full Model** | | | **Additive Model** | | |
| --- | --- | --- | --- | --- | --- | --- | --- | --- |
|  |  |  | **Estimate** | **SE** | **Power (%)** | **Estimate** | **SE** | **Power (%)** |
| 1_rs629301_T/G | *a* | 4.70 | 5.64 | 0.80 | 100 | 5.63 | 0.67 | 99 |
|  | *ae_4_* | 2.58 | 2.09 | 0.64 | 36 | 2.18 | 0.59 | 38 |
| 1_rs2499595_G/A | *a* | 1.54 | 1.69 | 0.41 | 28 | 1.67 | 0.39 | 42 |
|  | *ae_1_* | 1.43 | 2.10 | 0.51 | 19 | 1.98 | 0.53 | 26 |
|  | *ae_2_* | -3.61 | -3.89 | 1.00 | 29 | -3.43 | 0.97 | 42 |
|  | *ae_4_* | 2.03 | 2.41 | 0.43 | 21 | 2.39 | 0.47 | 29 |
| 2_rs478442_A/C | *a* | 4.02 | 4.65 | 0.92 | 65 | 4.25 | 0.50 | 67 |
| 2_rs5743063_G/A | *a* | -3.24 | -3.53 | 0.59 | 57 | -3.57 | 0.42 | 60 |
| 3_rs7624679_C/T | *a* | 1.10 | 2.09 | 0.57 | 46 | 1.89 | 0.53 | 65 |
|  | *ae_1_* | 1.56 | 1.92 | 0.41 | 20 | 1.89 | 0.46 | 19 |
|  | *ae_2_* | -2.85 | -3.24 | 1.37 | 22 | -3.12 | 0.99 | 31 |
|  | *ae_4_* | 2.07 | 2.48 | 0.62 | 15 | 2.28 | 0.56 | 24 |
| 5_rs17409624_G/A | *a* | 2.09 | 2.35 | 0.37 | 58 | 2.24 | 0.41 | 80 |
|  | *ae_3_* | 2.58 | 2.50 | 0.66 | 50 | 2.56 | 0.63 | 57 |
|  | *ae_4_* | -2.51 | -2.69 | 0.55 | 51 | -2.62 | 0.52 | 61 |
| 6_rs2609326_C/T | *a* | 2.16 | 2.64 | 0.27 | 33 | 2.51 | 0.34 | 52 |
| 7_rs213520_C/T | *a* | -1.73 | -2.63 | 0.35 | 11 | -2.35 | 0.36 | 28 |
| 8_rs10503377_C/T | *a* | 3.04 | 3.29 | 0.75 | 72 | 3.53 | 0.46 | 57 |
|  | *ae_1_* | -2.09 | -2.37 | 0.73 | 56 | -2.39 | 0.64 | 47 |
|  | *ae_2_* | 4.77 | 4.17 | 1.13 | 67 | 4.46 | 1.06 | 57 |
|  | *ae_4_* | -2.05 | -2.43 | 0.57 | 43 | -2.55 | 0.52 | 40 |
| 11_rs7124873_G/A | *a* | 2.75 | 3.05 | 0.47 | 73 | 2.96 | 0.45 | 87 |
|  | *ae_1_* | -1.47 | -1.84 | 0.55 | 36 | -1.83 | 0.57 | 45 |
|  | *ae_4_* | 1.68 | 2.23 | 0.55 | 27 | 2.22 | 0.51 | 28 |
| 13_rs942231_T/C | *a* | 2.03 | 2.50 | 0.28 | 35 | 2.35 | 0.27 | 59 |
| 13_rs1410165_G/A | *a* | 2.45 | 2.93 | 0.29 | 38 | 2.81 | 0.31 | 56 |
| 15_rs12595211_G/A | *a* | -2.76 | -3.11 | 0.52 | 51 | -3.09 | 0.46 | 50 |
| 16_rs1532625_G/A | *a* | -3.10 | -3.32 | 0.56 | 84 | -3.21 | 0.49 | 91 |
| 16_rs4888652_T/C | *a* | 2.34 | 2.26 | 0.33 | 29 | 2.24 | 0.30 | 42 |
|  | *ae_1_* | -1.39 | -1.89 | 0.52 | 10 | -1.73 | 0.42 | 14 |
| 17_rs2649485_C/T | *a* | -2.59 | -2.88 | 0.43 | 50 | -2.76 | 0.44 | 72 |
| 18_rs1105654_A/G | *a* | 2.32 | 2.86 | 0.35 | 28 | 2.81 | 0.27 | 36 |
| 19_rs1654452_C/T | *a* | -2.84 | -3.01 | 0.72 | 82 | -3.16 | 0.50 | 78 |
|  | *ae_3_* | -1.82 | -2.13 | 0.49 | 22 | -2.10 | 0.50 | 24 |
|  | *ae_4_* | 1.87 | 2.13 | 0.45 | 33 | 2.20 | 0.52 | 28 |

Chr_SNP_Allele: chromosome_QTS_major/minor alleles; Full Model: estimated or predicted effects from full model approach; Additive Model: estimated or predicted effects from multi-loci additive model approach; Effect: mode of gene action; Parameter: true genetic effects; Ave_Estimate: average estimated or predicted effects of genetic parameters; Power (%): power of detecting effects in percentage.

**Table S6. The power of detecting loci by different approaches under scenario-II**

| Chr_SNP_Alleles | Full Model | Additive Model | PLINK | GCTA |
| --- | --- | --- | --- | --- |
| 1_rs629301_T/G | 100 | 99 | 98 | 97 |
| 1_rs2499595_G/A | 28 | 49 | 4 | 2 |
| 2_rs478442_A/C | 65 | 67 | 58 | 52 |
| 2_rs5743063_G/A | 57 | 60 | 27 | 20 |
| 3_rs7624679_C/T | 61 | 68 | 2 | 2 |
| 5_rs17409624_G/A | 78 | 80 | 4 | 4 |
| 6_rs2609326_C/T | 43 | 52 | 5 | 4 |
| 7_rs213520_C/T | 21 | 28 | 2 | 1 |
| 8_rs10503377_C/T | 72 | 57 | 4 | 4 |
| 11_rs7124873_G/A | 73 | 87 | 9 | 8 |
| 13_rs942231_T/C | 45 | 59 | 2 | 1 |
| 13_rs1410165_G/A | 58 | 56 | 9 | 8 |
| 15_rs12595211_G/A | 51 | 50 | 14 | 10 |
| 16_rs1532625_G/A | 84 | 91 | 22 | 21 |
| 16_rs4888652_T/C | 39 | 42 | 2 | 1 |
| 17_rs2649485_C/T | 70 | 72 | 11 | 10 |
| 18_rs1105654_A/G | 38 | 36 | 0 | 0 |
| 19_rs1654452_C/T | 82 | 78 | 17 | 15 |

Chr_SNP_Alleles: chromosome_QTS_major/minor alleles; Full Model: Loci detection powers of full model, Additive Model: Loci detection powers of multi-loci additive model, PLINK: Loci detection powers of *PLINK* analysis, and GCTA: Loci detection powers of *GCTA* analysis.


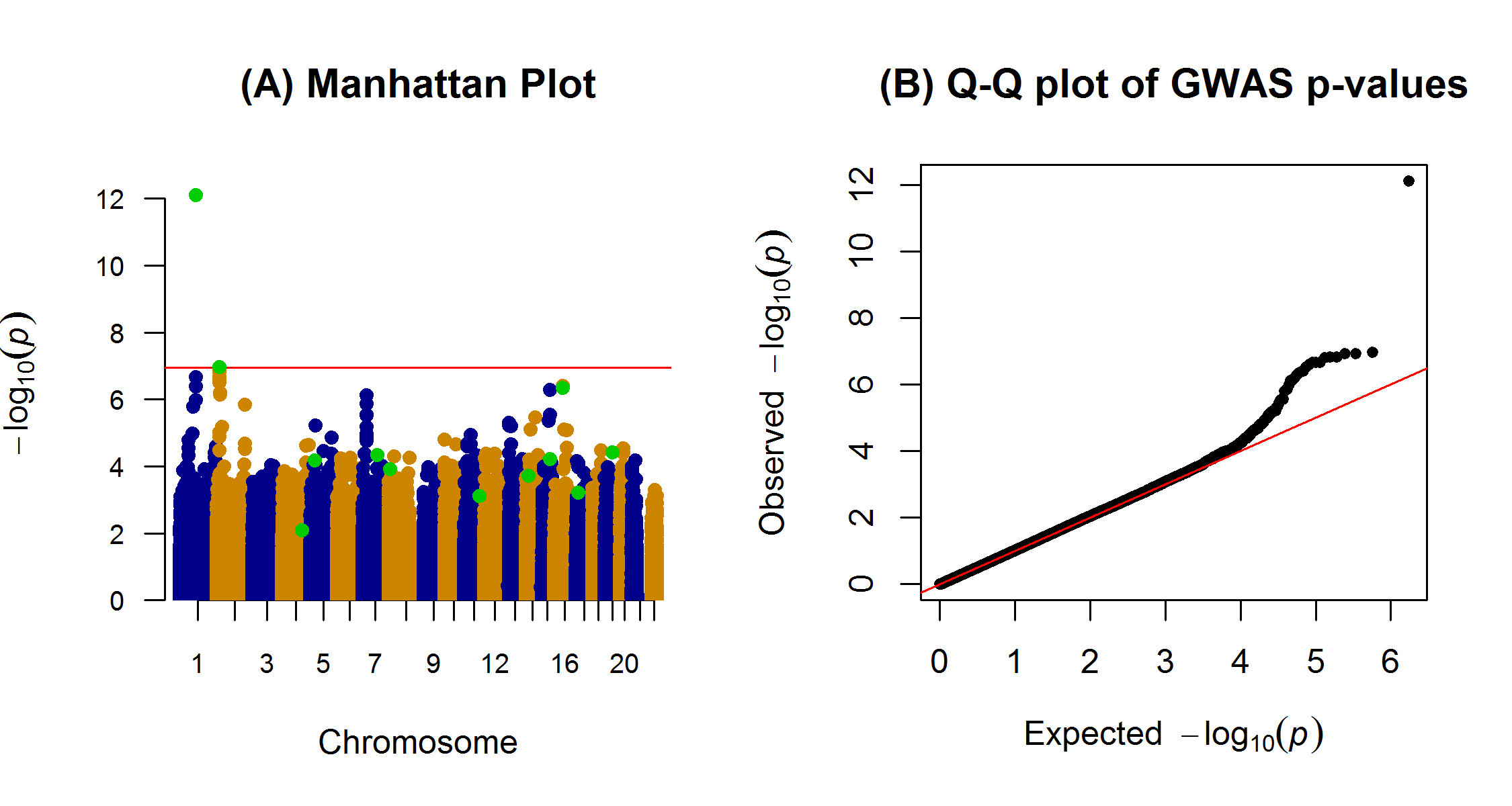


**Figure S1. PLINK analysis of cholesterol trait of MESA population for Examination 1**. (A) Manhattan plot of GWAS p-values; green dots represents minus log transformed p-values corresponding the SNPs that were detected by using Full model approach. (B) Q-Q plot of GWAS p-values for examination 1.


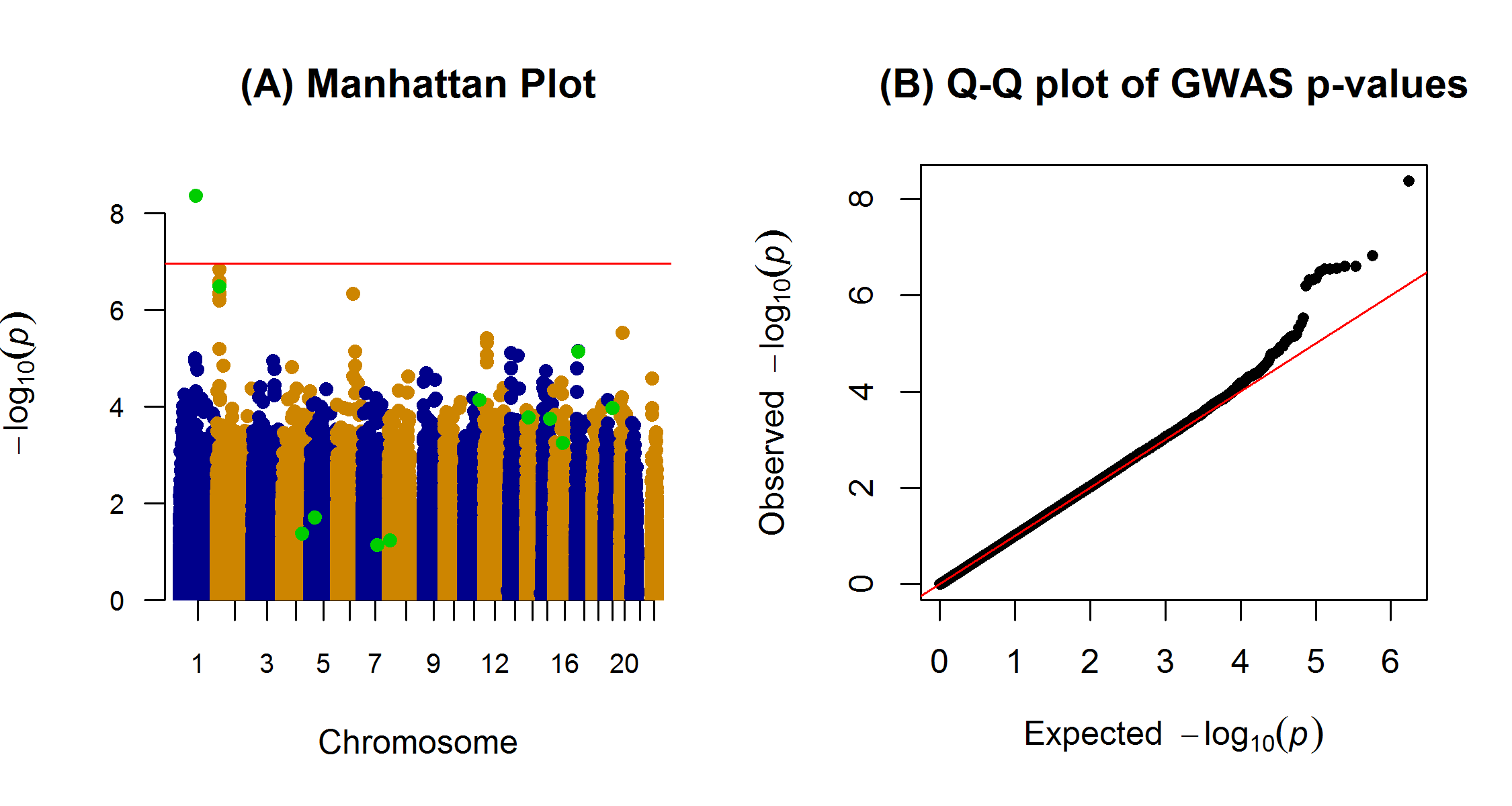


**Figure S2. PLINK analysis of cholesterol trait of MESA population Examination 3**. (A) Manhattan plot of GWAS p-values; green dots represents minus log transformed p-values corresponding the SNPs that were detected by using Full model approach. (B) Q-Q plot of GWAS p-values for examination 3.


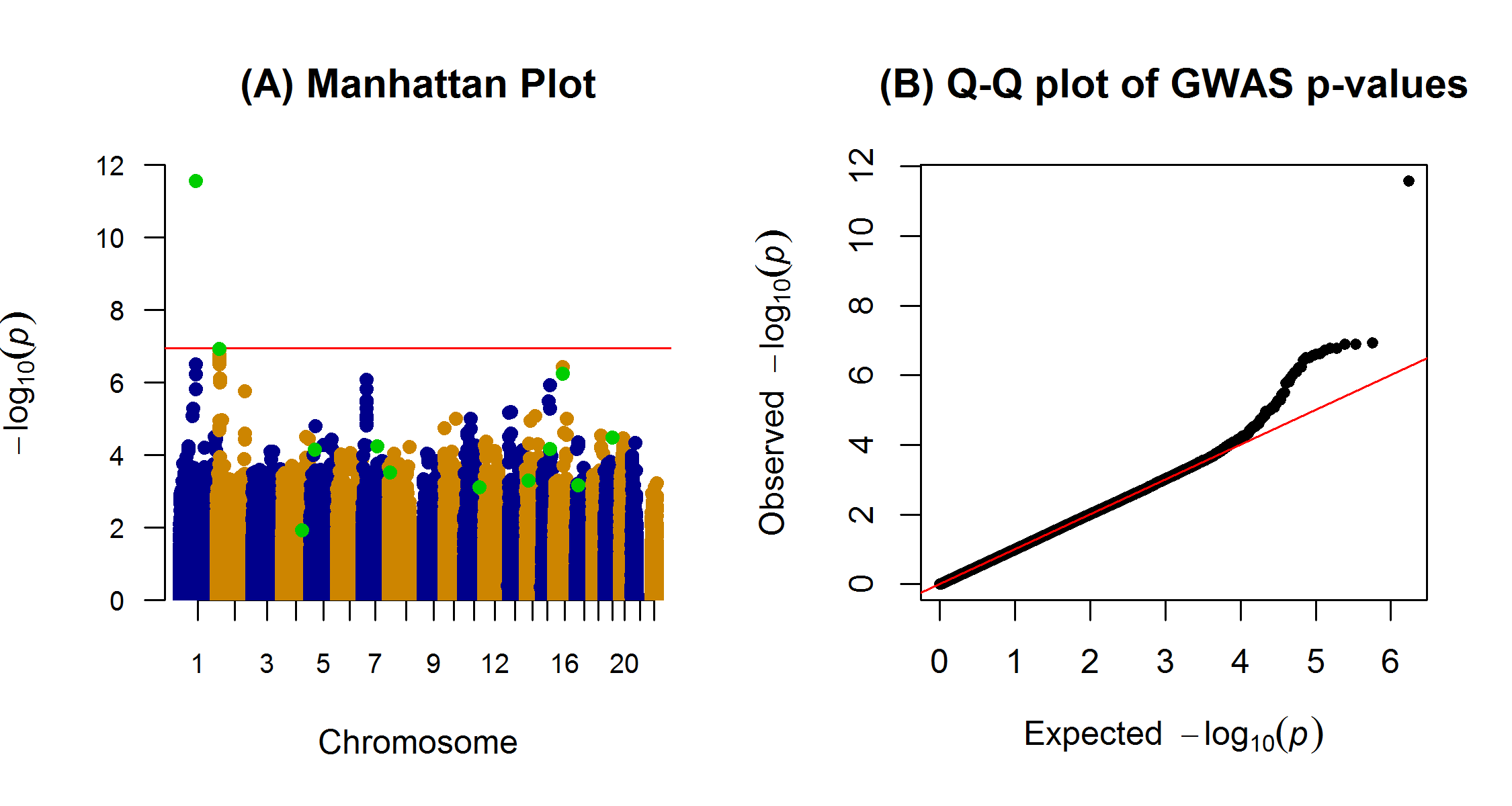


**Figure S3. GCTA analysis of cholesterol trait of MESA population for examination 1**. (A) Manhattan plot of GWAS p-values; green dots represents minus log transformed p-values corresponding the SNPs that were detected by using Full model approach. (B) Q-Q plot of GWAS p-values for examination 1.


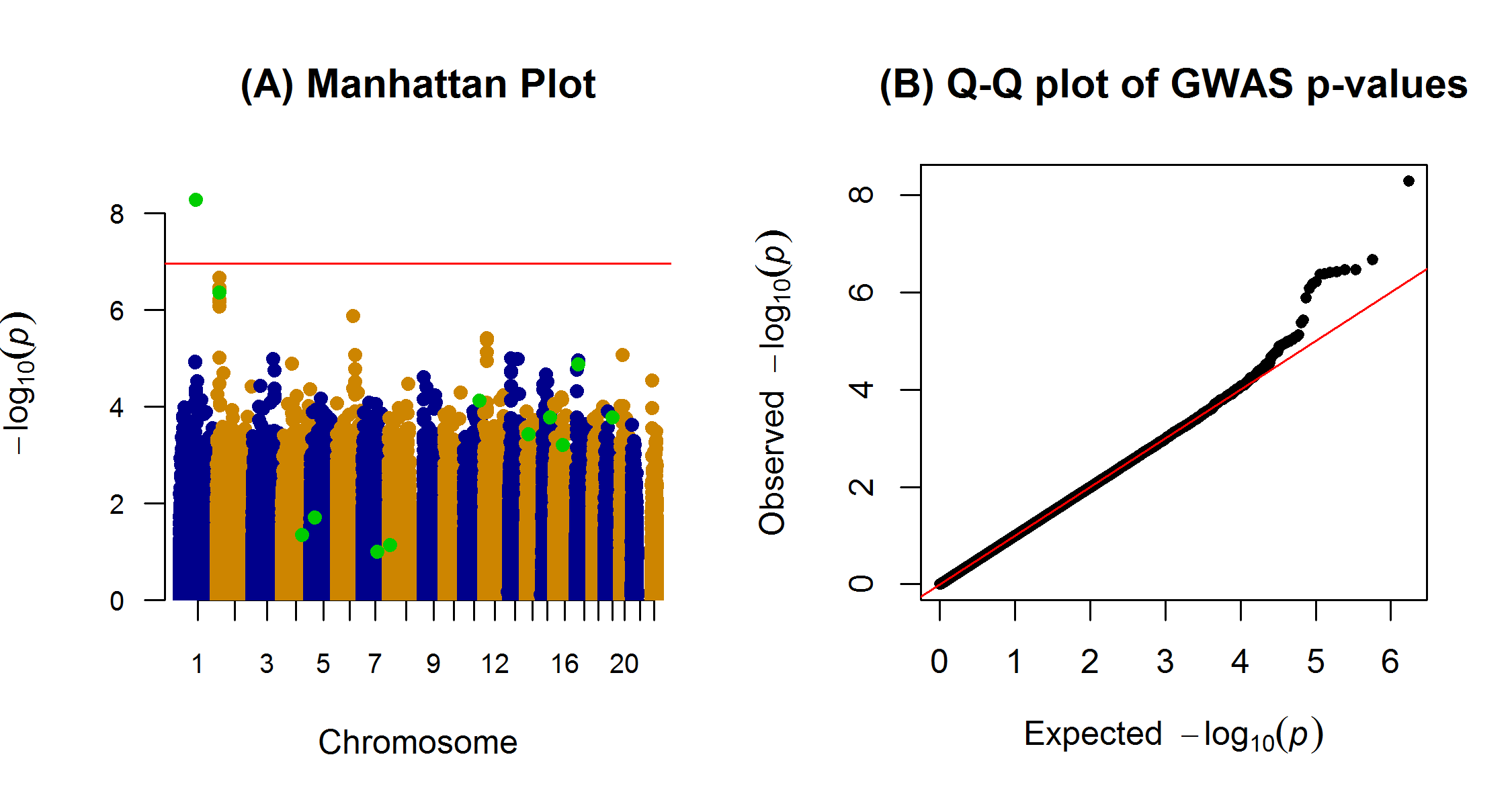


**Figure S4. GCTA analysis of cholesterol trait of MESA population for Examination 3**. (A) Manhattan plot of GWAS p-values; green dots represents minus log transformed p-values corresponding the SNPs that were detected by using Full model approach. (B) Q-Q plot of GWAS p-values for examination 3.

**
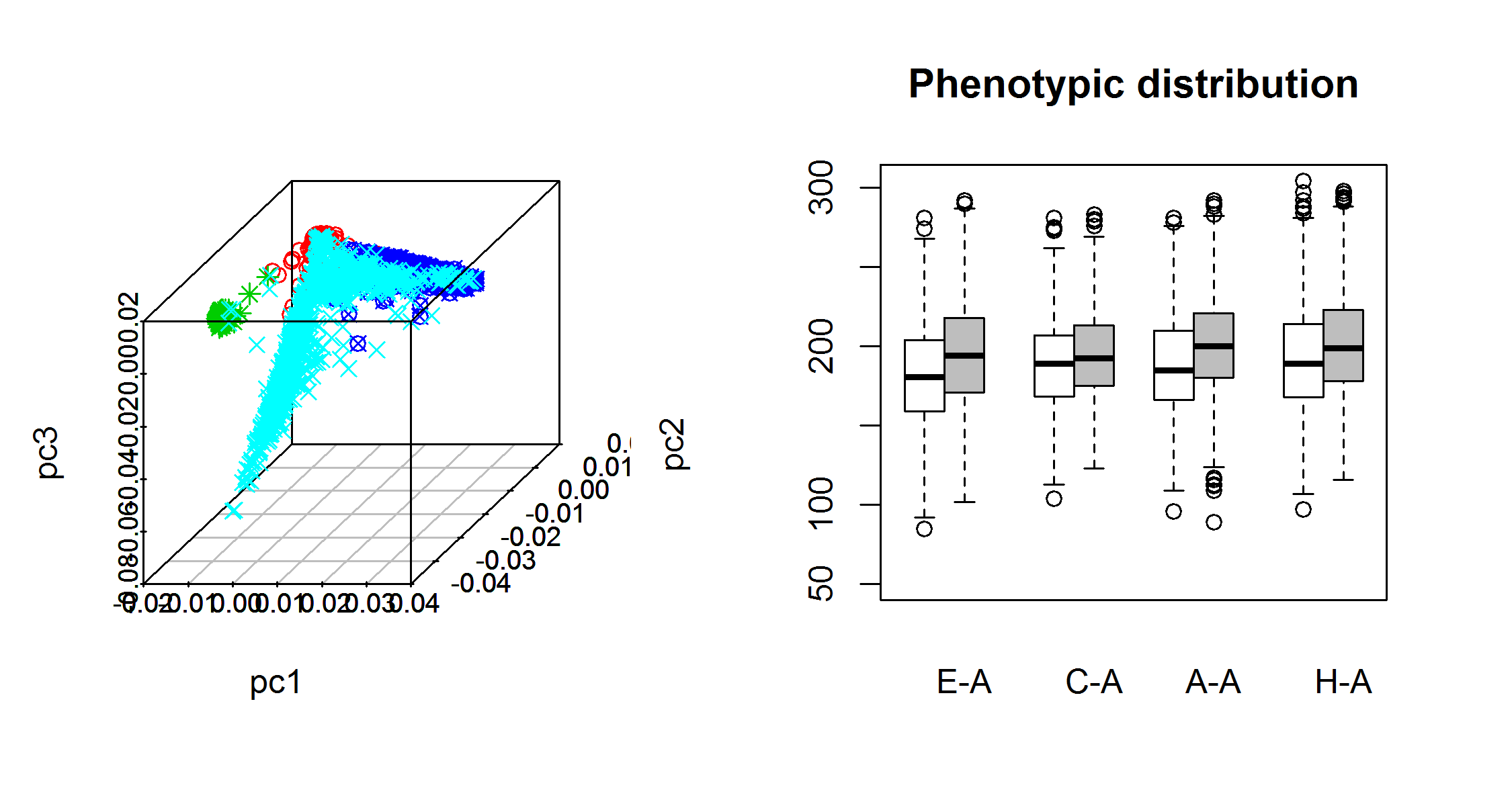
**

**Figure S5.** **Genotype and Phenotype distribution.** (A) Genotype distribution of four ethnic groups of MESA population; red-circle = European-Americans, green-star = Chinese-Americans, blue cross-circle = African-Americans, and sky-cross = Hispanic-Americans. (B) Ethnic and sex specific phenotypic distribution of MESA-population for Exam-1 data (July 2000-July 2002); White box for female and Gray box for male; *E-A* = European-Americans, *C-A* = Chinese-Americans, *A-A* = African-Americans, and *H-A* = Hispanic-Americans.
